# A rare combination of MODY5 and duodenal atresia in a patient: a case report

**DOI:** 10.1186/s12881-020-0954-0

**Published:** 2020-02-06

**Authors:** Tao Du, Nan Zeng, Xiaofang Wen, Peizhuang Zhu, Wangen Li

**Affiliations:** Department of Endocrinology, The Second Affiliated Hospital of Guangzhou Medical University, Guangzhou, 510260 People’s Republic of China

**Keywords:** 17q12 microdeletion, Duodenal atresia, Hepatocyte nuclear factor beta, B lymphocyte kinase

## Abstract

**Background:**

Maturity-onset diabetes of the young (MODY) is a genetically and clinically heterogeneous group of hereditary diabetes, generally caused by one abnormal gene. MODY5 is caused by mutations of the hepatocyte nuclear factor 1 homeobox β gene (HNF1β), always as a part of Chr17q12 deletion, whereas heterozygous mutation in B lymphocyte kinase (BLK) gene is responsible for MODY11.

**Case presentation:**

We report a patient who developed diabetes with a 1.58-Mb Chr17q12 microdeletion and BLK gene c.211G > A mutation using the cytoscan high-density array and whole-exome sequencing analysis. The patient received the surgery at five days after birth for the duodenal atresia and had normal growth postoperatively. Mild elevated liver enzymes were found along with the normal renal function. Quantitative analysis of β-cell function markers, including fasting insulin (< 0.2 mIU/L), fasting C-peptide (0.02 μg/L), postprandial-2 h insulin (< 0.2 mIU/L), and postprandial-2 h C-peptide (0.03 μg/L) suggested a severe loss of insulin secreting capacity. Meanwhile, islet autoantibodies (GADA, IA-2, ICA, and IAA) in the patient’s blood appeared negative. Neither dysplasia in other tissues nor abnormality in development and behavior was found.

**Conclusion:**

To date, gastrointestinal malformations were extremely rarely reported in patients with MODY. Our clinical report further expands the clinical presentation and variability of MODY5.

## Background

Maturity-onset diabetes of the young (MODY) is a monogenic form of non-autoimmune diabetes mellitus that usually first presents in adolescence or young adulthood, accounting for up to 2% of diabetes in people ages 20 and younger in the United States [1]. Depending on the clinical and genetic heterogeneity, 14 subtypes of genetic defects have been proposed to cause MODY [2]. For example, mutation in hepatocyte nuclear transcription factor 1 homeobox β (HNF1β) causes MODY5, which accounts for 2 to 6% of MODY [3]. On the other hand, approximately 50% of patients with MODY5 are diagnosed with a whole HNF1β gene deletion, which is virtually always part of a Chr17q12 deletion syndrome. Chr17q12 deletion syndrome usually comprises MODY5, and other diseases (i.e., cystic renal disease, pancreatic atrophy, female genital tract abnormalities, and variable cognitive involvement) [4, 5]. MODY11 is caused by Ala71Thr mutation in the B lymphocyte kinase (BLK) gene with attenuated BLK activity [6]. BLK is expressed in β-cells and enhances insulin synthesis and secretion in response to glucose.

Up to now, there is only one case with duodenal atresia reported to be associated with a 17q12 microdeletion [7]. Here, we report a MODY patient, with Chr17q12 microdeletion and BLK gene c.211G > A mutation, who had surgery at 5 days after birth for the duodenal atresia. The patient presented bilateral renal cyst without other illnesses or health problems.

## Case presentation

The patient was born after a healthy gestation in the Guangdong Province of China and received surgery at 5 days after birth for intestine obstruction caused by the duodenal atresia. The patient’s parents were nonconsanguineous and healthy without a family history of genetic disorders, congenital malformations, diabetes, and psychiatric disease. The patient was diagnosed with diabetes in a medical checkup at the age of 27 years in 2011. Since then, the patient has been receiving insulin treatment and had two pregnancies. During her first pregnancy in 2015, the patient was found with the fetal renal dysplasia and 17q12 deletion (34,822,465-36,283,612) through by B-mode ultrasound and umbilical cord blood gene test, resulting in an induced abortion. Consequently, the patient carried a successful pregnancy and had a healthy daughter in 2017.

In March 2018, the patient was re-hospitalized due to poor glycemic control and hyperketonemia. Anthropometric measurements showed the patient had a body weight of 52 kg (average body weight of a Chinese female is 57.3 kg), a height of 162 cm (average height of a Chinese female is 155.8 cm), and a body mass index (BMI) of 19.81 kg/m^2^ (normal rang 18.5–23.9 kg/m^2^). Other physical examination results were likewise unremarkable. Mild elevated liver enzymes were discovered (aspartate aminotransferase 47 IU/L, alanine aminotransferase 52 IU/L, lactose dehydrogenase 518 IU/L, alkaline phosphatase 152 IU/L and γ-glutamyl transferase 54 IU/L), while renal function was normal (blood urea nitrogen 6.07 mmol/L, creatinine 61.1 μmol/L, negative for urinary protein). Plasma glucose was 16.74 mmol/L, HbA1c was 12.2%, and blood ketone was 2.8 mmol/L. Analysis of β-cell function markers, including fasting insulin (< 0.2 mIU/L), fasting C-peptide (0.02 μg/L), postprandial-2 h insulin (< 0.2 mIU/L), and postprandial-2 h C-peptide (0.03 μg/L) suggested a severe loss of insulin secreting capacity (electrochemiluminescence immunoassay was used for insulin and C-peptide concentration). The patient was treated with insulin aspart in continuous insulin infusion (20/U insulin for basal/pre-meal bolus doses). Lab examination results showed negative anti-islet autoantibodies (GADA, IA-2, ICA, and IAA) in the patient’s blood using ELISA (GADA), radiobinding assay (IA-2), and radioimmunoassay (ICA and IAA), respectively. Echocardiography and plain X-rays analysis showed normal results. B-mode ultrasound showed a bilateral renal cyst, and cardiac color doppler ultrasound displayed a slightly tricuspid regurgitation.

**Fig. 1:**
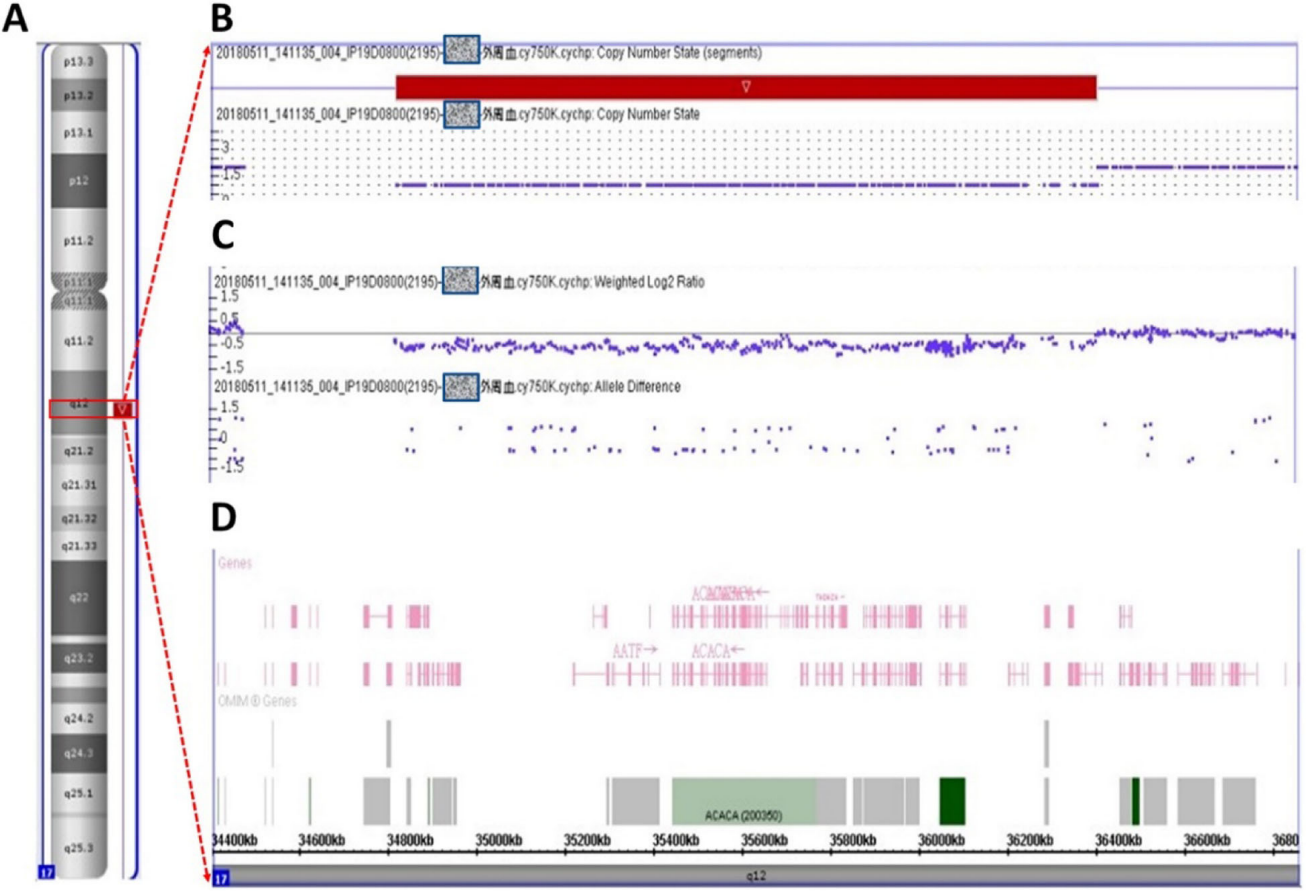
Molecular karyotyping revealed a deletion of approximately 1.58-Mb in Chr17q12 in patients. **a** Chromosome ideogram. The deletion region is highlighted in red. **b** Cytoscan high-density array identified a 1.58-Mb region (34,822,465-36,404,104) of hemizygous loss. **c** Magnification of the hemizygous loss region. **d** Genes located in the 1.58-Mb hemizygous loss region

**Fig. 2:**
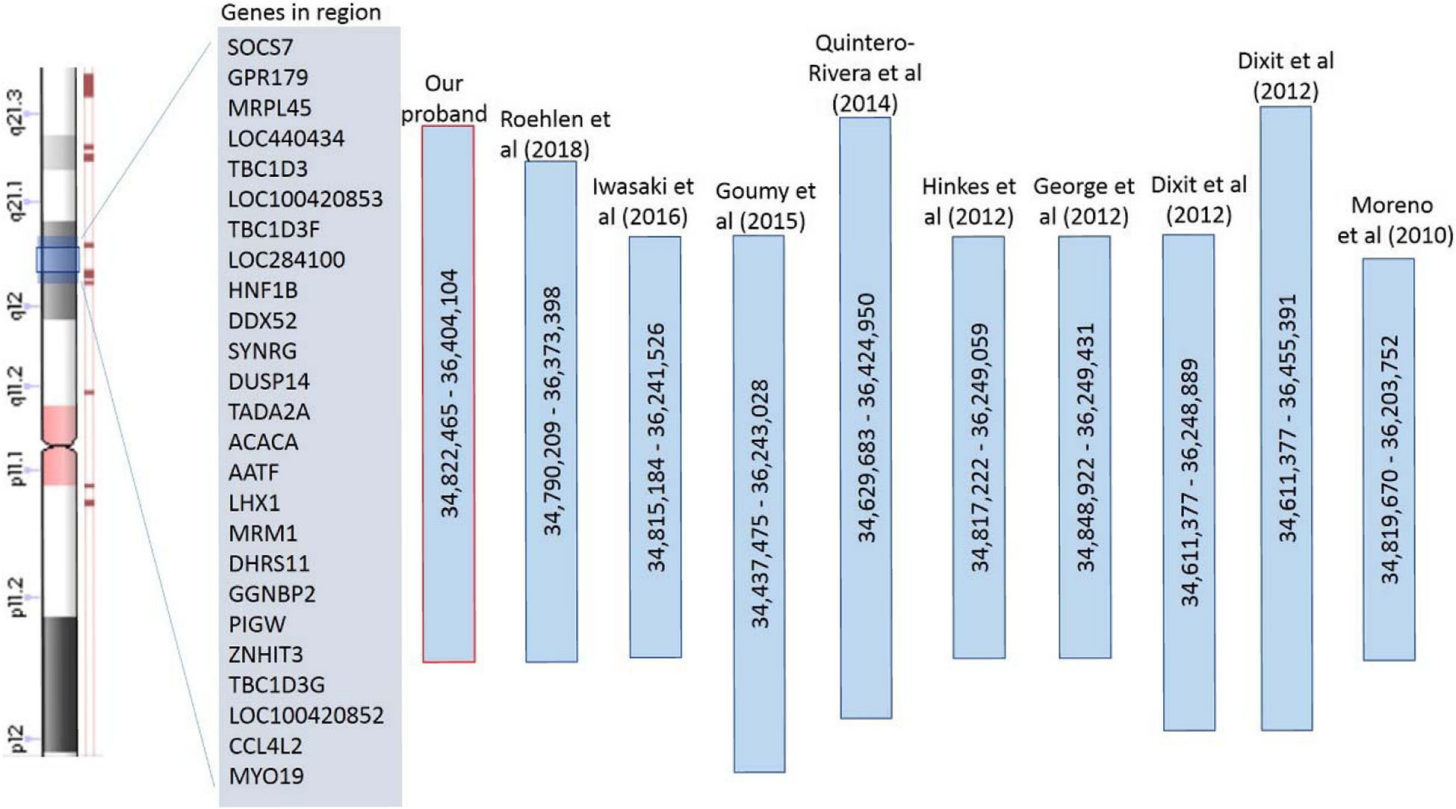
Comparison of deleted regions of Chr17q12. All genomic coordinates were converted to GRCh37/hg19 for comparison

To pinpoint the causative mutations in the patient, we used the cytoscan high-density array to identify microduplications or microdeletions, and used whole-exome sequencing to detect the mutations in genes. In brief, the cytoscan 750 K Array (Affymetrix Inc., Santa Clara, CA) was used for chromosomal microarray, containing 550,000 nonpolymorphic probes and 200,436 single nucleotide polymorphic probes. The copy number variation was analyzed using the Affymetrix Chromosome Analysis Suite software (Version 3.0) and interpreted with the aid of UCSC genome browser (http://gemome.ucsc.edu). The exon coding regions of ABCC8, AKT2, BLK, CEL, EIF2AK3, GCK, GLIS3, GLUD1, HADH, HNF1A, HNF1B, HNF4A, INS, INSR, KCNJ11, KLF11, MAPK8IP1, NEUROD1, PAX4, PDX1, PLAGL1, PTF1A, RFX6, SLC19A2, SLC2A2, UCP2, and ZFP57 were directly sequenced to discover possible gene mutations. The analysis results showed a 1.58-Mb region (34,822,465-36,404,104) of hemizygous loss in Chr17q12 (Fig. 1) and BLK gene p.Ala71Thr (c.211G > A) mutation. The patient showed diabetes and renal cysts. Nevertheless, no other illnesses or health problems were found. To elucidate the various clinical presentations and characteristics of Chr17 deletion, we compared the clinical manifestations in our proband and previously reported patients with a similar deletion region (34,437.475-36,424,950) in Chr17q12 [7–15] (Fig. 2, Table 1). As shown in Table 1, individuals with similar gene deletions could manifest various presentations and disorders (such as diabetes, renal cysts, liver and pancreas malformations, and intellectual disability). Even between parents and children without differences in the deletion region, the significant variance in clinical phenotypes is presented [7, 11, 13]. Likewise, the severity of renal and pancreatic defects varied between monozygotic twins with MODY5 due to Chr17q12 deletion [8].

**Table 1:** Comparisons of clinical manifestations in our proband and previously reported patients with a similar phenotype or deletion region in Chr17q12

	Our proband	Roehlen et al. (2018) [15]	Iwasaki et al. (2016) [9]	Goumy et al. (2015) [14]	Quintero-Rivera et al. (2014) [7]	Hinkes et al. (2012) [12]	George et al. (2012) [11]	Dixit et al. (2012) [10]	Moreno-De-Luca et al. (2010) [13]
Diabetes mellitus	+	2/2	+	NR	–	+	0/2	0/3	1/9
Renal cysts	+	2/2	+	+	+	+	1/2	3/3	+
Pancreas abnormalities	–	2/2	+	NR	–	NR	NR	0/3	NR
Liver abnormalities	+	2/2	+	+	+	NR	NR	1/3	NR
Facial dysmorphism	–	1/2	NR	+	+	NR	NR	2/3	9/9
Joint laxity	–	NR	NR	NR	+	+	NR	0/3	NR
Short stature or failure to thrive	–	NR	NR	–	–	NR	0/2	1/3	1/9
Autism spectrum disorder	–	NR	NR	–	NR	–	0/2	1/3	6/9
Intellectual impairment	–	2/2	NR	–	NR	–	2/2	3/3	8/9
Aggression	–	NR	NR		NR	–	NR	NR	2/9
Anxiety/disruptive behavior	–	NR	NR	–	NR	–	1/2	NR	5/9
Hyperactivity	–	NR	NR	NR	–	–	1/2	NR	2/9
Gastrointestinal abnormalities	DA	NR	NR	GERD	DA	NR	NR	NR	GERD (2/9), FC (2/9)

+: feature is present, −: feature is absent, *NR* Not reported, *DA* Duodenal atresia, *GERD* Gastroesophageal reflux disease, *FC* Frequent constipation

## Discussion and conclusions

It has been reported that the incidence of MODY5 is up to 50% [16] or 82% in Chr17q12 deletion [17]. The renal cystic dysplasia and other structural renal anomalies are the most commonly reported manifestation of the Chr17q12 deletion [16, 17]. The whole gene deletion and mutations in the coding region or splice sites of HNF1B cause MODY5 (2 to 6% of MODY diagnoses) that is typically characterized by additional HNF1B-related kidney disease, pancreatic hypoplasia, genital tract malformations, abnormal liver function and early-onset gout [3]. It is known that the minimum size for the Chr17q12 deletion is 1.4 Mb DNA sequence in the approximate position of chr17: 34,815,072 - 36,192,492 [16]. Our patient’s deletion is 1.58-Mb DNA sequence at the position of chr17: 34,822,465-36,404,104. Notably, our patient diagnosed as MODY5 with bilateral renal cysts has no other severe abnormal phenotypes, while some patients with similar deletion region of chr17 were reported to have the short stature, speech delay and dyspraxia, psychopathology, or autistic traits (Table 1). Among previously studies, three studies reported the gastroesophageal reflux disease in individuals with chr17q12 deletion [13, 14, 18], and only one reported the duodenal atresia in the individual with chr17q12 deletion [7]. Interestingly, duodenal atresia has been reported in one patient with 17q12 duplication [18]. Therefore, it is strongly suggested that genetic defects alone are not sufficient to result in the clinical features associated with the Chr17q12 deletion.

In our patient, BLK Ala71Thr mutation was detected. In 2004, Kim et al. firstly reported the MODY was associated with Chr8p23 [19]. Borowiec et al. showed the BLK Ala71Thr mutation was responsible for MODY11 by resequencing genomic sequence at 8p23 in 6 MODY families, and revealed the mutation altered the BLK expression and affected MIN6 B-cells activity [6]. However, Bonnefond et al. demonstrated a nominal association between this variant and increased type 2 diabetes risk and no mutation in BLK in 64 unelucidated MODY samples [20]. The genome databases also show the frequency of BLK p.Ala71Thr (c.211G > A) mutation is very low, only 0.01193 from the Genome Aggregation Database (gnomAD), 0.01171 from Exome Aggregation Consortium (ExAC), and 0.01238 from Global Minor Allele Frequency (GMAF). It suggests that the BLK variant is a benign or a pathological factor combined with other diabetic risk factors. In our case, the patient had a severe loss of insulin secretion capacity with BLK mutation and Chr17q12 deletion, but it was still difficult to evaluate the effect of BLK mutation on diabetes. According to the previously reported data, the BLK variant as a pathology of MODY still requires further validation.

The previous studies revealed the various clinical manifestations of Chr17q12 deletion. Here, we reported a woman who developed MODY5 with the Chr17q12 deletion and duodenal atresia. Our reported new phenomena further demonstrated the clinical variability of MODY5.

## Data Availability

The data used and/or analyzed in the present report were deposited in the Sequence Read Archive (SRA) database. The data are accessible via the SRA accession: PRJNA600784; or via the links: https://www.ncbi.nlm.nih.gov/sra/PRJNA600784; https://trace.ncbi.nlm.nih.gov/Traces/sra/?run=SRR10875069.

## Abbreviations

ABCC8: Adenosine triphosphate (ATP)-binding cassette; sub-family C (CFTR/MRP) member 8
AKT2: Protein kinase B beta
BLK: Heterozygous mutation in B lymphocyte kinase
BMI: Body mass index
CEL: Carboxyl ester lipase
EIF2AK3: Eukaryotic translation initiation factor 2-alpha kinase 3
ELISA: Enzyme-linked immunosorbent assay
ExAC: Exome Aggregation Consortium
GADA: Glutamic acid decarboxylase 65 autoantibody
GCK: Glucokinase (GCK)
GLIS3: GLI-similar 3
GLUD1: Glutamate dehydrogenase 1 gene
GMAF: Global Minor Allele Frequency
gnomAD: Genome Aggregation Database
HADH: Hydroxy-acyl-CoA dehydrogenase
HNF1A: Hepatocyte nuclear factor 1A
HNF1B: Hepatocyte nuclear factor 1B
HNF1β: Hepatocyte nuclear factor 1 homeobox β gene
HNF4A: Hepatocyte nuclear factor 4A
IA2A: Insulinoma-associated protein 2 autoantibody
IAA: Insulin autoantibody
ICA: Islet cell autoantibodies
INS: Insulin
INSR: Insulin receptor
KCNJ11: Potassium channel inwardly rectifying subfamily; member 11
KLF11: Kruppel-like factor 11
MAPK8IP1: Mitogen-activated protein kinase 8 interacting protein 1
MODY: Maturity-onset diabetes of the young
NEUROD1: Neurogenic differentiation 1
PAX4: Paired-box-containing gene 4
PDX1: Pancreatic and duodenal homeobox 1
PLAGL1: Pleiomorphic adenoma gene 1
PTF1A: Pancreas-specific transcription factor 1A
RFX6: Regulatory factor X 6
SLC19A2: Solute carrier family 19 member 2
SLC2A2: Solute carrier family 2 member 2
UCP2: Uncoupling Protein 2
ZFP57: Zinc finger protein 57
